# Association of antioxidant markers (SOD, GSH, NO, Catalase) with ischemic stroke severity and recovery outcomes

**DOI:** 10.5937/jomb0-57208

**Published:** 2025-07-04

**Authors:** Chen Haonan, Zang Shuhan, Zhang Runlei, Chen Peilin, Wu Shengxian

**Affiliations:** 1 Dongzhimen Hospital, Beijing University of Chinese Medicine, Beijing, China; 2 Peking University International Hospital, Beijing, China

**Keywords:** ischemic stroke, antioxidant markers, superoxide dismutase, glutathione, catalase, nitric oxide, stroke severity, recovery outcome, ishemijski moždani udar, antioksidativni markeri, superoksid dismutaza, glutation, katalaza, azotmonoksid, težina moždanog udara, oporavak

## Abstract

**Background:**

This randomised controlled trial aimed to investigate the relationship between antioxidant markers (Superoxide Dismutase [SOD], Glutathione [GSH], Catalase, and Nitric Oxide [NO]) and the severity of ischemic stroke in affected individuals.

**Methods:**

A single-blind randomised controlled trial was conducted from June 2022 to November 2024, including 364 patients aged 45-80 years diagnosed with ischemic stroke. Participants were randomly divided into two groups: Group A (n=193) received standard stroke rehabilitation therapy, while Group B (n=171) received additional antioxidant support. Serum levels of SOD, GSH, Catalase, and NO were measured. Stroke severity was evaluated using the modified Rankin Scale (mRS) and NIH Stroke Scale (NIHSS), with follow-up assessments at 2, 4, and 6 months post-treatment.

**Results:**

Among the 364 participants, 203 (55.7%) were male, and 161 (44.3%) were female, with a mean age of 67.3±12.2 years. Serum SOD levels were higher in the experimental group (16.3±3.7 U/mL) compared to the control group (12.5±4.1 U/mL, p=0.014). GSH levels were also significantly higher in the experimental group (178±31 mmol/L) than in the control group (145±26 mmol/L, p=0.032). NO levels were higher in the experimental group (42.1±8.6 mmol/L) than in the control group (35.4±7.3 mmol/L, p=0.021). Catalase levels were 52.3±11.1 U/mL in the experimental group and 49.6±10.2 U/mL in the control group, with no significant difference between the groups (p=0.213). Significant inverse correlations were found between SOD, GSH, and NO levels and stroke severity (p<0.05), but catalase showed no such correlation (p=0.513).

**Conclusions:**

This study identified a significant relationship between higher levels of SOD, GSH, and NO and improved stroke recovery, whereas catalase did not exhibit a meaningful association with stroke severity or functional outcomes. These findings highlight the potential role of specific antioxidant markers in stroke prognosis and recovery while suggesting that catalase may not play a critical role in ischemic stroke outcomes.

## Abstract (sr)

**Uvod:**

Cilj ove randomizovane kontrolisane studije bio je da ispita povezanost antioksidativnih markera - superoksid dismutaze (SOD), glutationa (GSH), katalaze i azot-monoksida (NO) - sa težinom ishemijskog moždanog udara kod obolelih pacijenata.

**Metode:**

Jednostruko "slepa" i randomizovana kontrolisana studija sprovedena u periodu od juna 2022. do novembra 2024. godine, a obuhvatila je 364 pacijenta, starosti 45-80 godina, sa dijagnozom ishemijskog moždanog udara. Ispitanici su nasumično podeljeni u dve grupe: grupa A (n=193) primala je standardnu rehabilitacionu terapiju, dok je grupa B (n=171) dobijala dodatnu antioksidativnu podr{ku. Nivoi SOD-a, GSH-a, katalaze i NO-a određivani su u serumu. Težina moždanog udara procenjivana je pomo}u modifikovane Rankinove skale (mRS) i NIH skale za moždani udar (NIHSS). Kontrolna merenja su vršena nakon 2, 4 i 6 meseci od početka terapije.

**Rezultati:**

Od ukupno 364 pacijenta, 203 (55,7%) su bila muškog pola, a 161 (44,3%) ženskog, sa prosečnom starošću od 67,3±12,2 godine. Nivoi SOD-a bili su značajno viši u eksperimentalnoj grupi (16,3±3,7 U/mL) u poređenju sa kontrolnom (12,5±4,1 U/mL; p=0,014). Takođe, nivoi GSH-a su bili viši u eksperimentalnoj grupi (178±31 mmol/L) u odnosu na kontrolnu (145±26 mmol/L; p=0,032). Nivoi NO-a bili su povećani u eksperimentalnoj grupi (42,1±8,6 mmol/L) u poređenju sa kontrolnom (35,4±7,3 mmol/L; p=0,021). Nije zabeležena značajna razlika u nivoima katalaze između grupa (52,3±11,1 U/mL naspram 49,6±10,2 U/mL; p=0,213). Uočene su značajne inverzne korelacije između nivoa SOD-a, GSH-a i NO-a i težine moždanog udara (p<0,05), dok katalaza nije pokazala statistički značajnu povezanost (p=0,513).

**Zaključak:**

Studija je pokazala značajnu povezanost viših nivoa SOD-a, GSH-a i NO-a sa boljim ishodima oporavka kod pacijenata sa ishemijskim moždanim udarom, dok katalaza nije pokazala značajnu povezanost sa težinom udara niti funkcionalnim ishodima. Dobijeni rezultati ukazuju na potencijalnu prognostičku vrednost odre|enih antioksidativnih markera u proceni težine i oporavka od ishemijskog moždanog udara.

## Introduction

Ischemic stroke, a condition resulting from the obstruction of blood flow to the brain, continues to be one of the most significant causes of morbidity and mortality worldwide [1]. Stroke is characterised by the sudden cessation of blood supply to specific regions of the brain, leading to a cascade of pathophysiological events, including neuronal injury, inflammation, and oxidative stress [2] [3]. The damage caused by ischemic stroke can range from mild deficits to severe disability, depending on factors such as the size and location of the infarct, the time to medical intervention, and the underlying health status of the individual [4]. Given the profound impact ischemic stroke has on patients and their families, understanding the factors that contribute to stroke severity and recovery is crucial for improving treatment outcomes and developing targeted interventions.

Oxidative stress, which occurs when the production of reactive oxygen species (ROS) exceeds the body’s ability to neutralise them with antioxidants, is a critical pathological feature of ischemic stroke [5] [6]. The brain, with its high metabolic rate and relatively low antioxidant capacity, is particularly vulnerable to the damaging effects of ROS [7] [8]. These free radicals can damage cellular structures, including lipids, proteins, and DNA, further exacerbating neuronal injury and contributing to brain dysfunction [9]. In this regard, antioxidants play a vital role in defending the brain against oxidative damage and promoting cellular repair processes. Key antioxidant markers such as Superoxide Dismutase (SOD), Glutathione (GSH), Catalase, and Nitric Oxide (NO) have garnered increasing attention for their potential role in mitigating the effects of oxidative stress in stroke [10].

Previous studies have explored the role of these antioxidants in ischemic stroke, with varying results. SOD is one of the primary enzymes responsible for converting superoxide radicals into hydrogen peroxide, which is then further detoxified by catalase and GSH [11]. Studies have found that higher SOD levels correlate with better functional recovery, suggesting its protective role in stroke outcomes [12] [13]. GSH, a major intracellular antioxidant, works by directly neutralising ROS and by participating in various cellular protective processes [14]. Clinical studies indicate that elevated GSH levels are linked to better recovery, reinforcing its neuroprotective function [15]. Catalase, an enzyme found in nearly all aerobic organisms, plays a central role in converting hydrogen peroxide into water and oxygen, thereby preventing the accumulation of harmful oxidative byproducts [16]. NO, though primarily recognised for its vasodilatory properties, also acts as an antioxidant in the brain, potentially protecting neurons from ischemia-induced damage through various mechanisms, including modulating blood flow and reducing neuronal apoptosis [17]. Studies reported that higher NO levels were associated with better functional recovery, but conflicting findings suggest its effects may be dose-dependent [18].

Despite the central role these antioxidants play in the brain’s defence against oxidative damage, the relationship between their serum levels and the clinical outcomes of ischemic stroke remains poorly understood. Previous studies have indicated varying degrees of association between antioxidant levels and stroke severity, with some suggesting that higher antioxidant levels correlate with better recovery outcomes, while others have failed to demonstrate consistent patterns [19] [20] [21] [22]. Furthermore, while the role of certain antioxidants, such as SOD and GSH, in ischemic injury has been well-documented in animal models, human studies have yielded mixed results, highlighting the need for further investigation in clinical populations.

This study seeks to address the gap in knowledge by exploring the relationship between antioxidant markers – specifically SOD, GSH, Catalase, and NO – and ischemic stroke severity, as well as the potential for recovery in human subjects. Given the conflicting findings in previous research, a key goal of this study is to clarify the role of these antioxidants as biomarkers for stroke severity and functional outcomes. We hypothesise that higher levels of SOD, GSH, and NO will be associated with less severe stroke outcomes and better recovery, while lower levels will correlate with more severe impairment and poorer rehabilitation prospects. By examining these markers in a large cohort of ischemic stroke patients, this study aims to clarify their role in the pathophysiology of ischemic stroke and provide insights into their potential as biomarkers for stroke prognosis and therapeutic targets.

Furthermore, this research aims to assess whether antioxidant supplementation may enhance stroke recovery. Participants were randomly divided into two groups: one receiving standard rehabilitation therapy and the other receiving additional antioxidant support. This design allows for a deeper understanding of how antioxidant supplementation may influence stroke recovery beyond its role in reducing oxidative stress. Ultimately, the goal of this study is not only to identify key antioxidant markers associated with stroke severity but also to contribute to the development of more personalised and effective therapeutic strategies in stroke management.

## Materials and methods

### Study design

This study was a randomised controlled, single-blind clinical trial conducted from June 2022 to November 2024. The primary objective was to assess the association between antioxidant markers (Superoxide Dismutase [SOD], Glutathione [GSH], Catalase, and Nitric Oxide [NO]) and the severity of ischemic stroke and recovery outcomes. The study was approved by the institutional review board (IRB) of [Institution Name], and all participants provided written informed consent prior to enrollment.

### Study population

The study included 364 adult patients aged 45-80 years who were diagnosed with ischemic stroke based on clinical and radiological criteria (CT or MRI confirming ischemia) within 72 hours of symptom onset. Participants were recruited and were screened for eligibility between June 2022 and June 2023.

Inclusion criteria included:

- A clinical diagnosis of ischemic stroke, confirmed by CT or MRI
- Age between 45 and 80 years
- Ability to provide informed consent

Exclusion criteria included:

- Hemorrhagic stroke
- Severe comorbid conditions such as cancer, severe liver or kidney disease, or terminal illness
- Pregnancy or lactation
- Pre-existing neurological disorders (e.g., dementia, Parkinson’s disease)
- Recent use of antioxidants or drugs affecting antioxidant metabolism

Of the 442 patients initially screened, 78 patients were excluded based on the following reasons:

- 34 patients (43.6%) had a history of hemorrhagic stroke,
- 19 patients (24.4%) had severe comorbidities that met exclusion criteria,
- 11 patients (14.1%) had pre-existing neurological disorders,
- 8 patients (10.3%) had been taking antioxidant supplements prior to enrollment,
- 6 patients (7.7%) were unable to provide informed consent due to cognitive impairment or communication difficulties.

This exclusion process ensured that the final study population was homogeneous and free from confounding factors that might influence oxidative stress and stroke recovery outcomes, thereby improving the internal validity of the study.

### Randomisation and group allocation

Participants were randomly assigned to one of two treatment groups:

- Group A (Control Group, n=193): Received standard stroke rehabilitation therapy, including physical therapy, occupational therapy, and speech therapy, tailored to their individual deficits.
- Group B (Experimental Group, n=171): Received standard stroke rehabilitation therapy in addition to antioxidant supplementation (details below).

Randomisation was performed using a computer-generated random number table, ensuring balanced allocation across both groups. An independent research coordinator conducted the randomisation process, and both the research team and participants were blinded to group assignments.

### Antioxidant supplementation

Participants in Group B received a combination of antioxidant supplements for 6 months post-stroke. The specific dosages of SOD, GSH, Catalase, and NO precursor (L-arginine) were selected based on previous clinical studies and established guidelines on antioxidant therapy in ischemic stroke and oxidative stress-related conditions. The supplementation regimen was as follows:

- SOD (Superoxide Dismutase): 50 mg daily
- This dosage was selected based on studies indicating that 50 mg/day of oral SOD improves antioxidant defence and reduces oxidative damage in ischemic stroke models.
- GSH (Glutathione): 500 mg daily
- Clinical studies have suggested that oral glutathione supplementation at doses of 500 mg/day enhances cellular antioxidant capacity and promotes neuroprotection in stroke patients.
- Catalase: 250 U daily
- This dosage was chosen based on its known role in hydrogen peroxide detoxification. Previous research suggests that 250 U/day is sufficient to enhance antioxidant defence without adverse effects.
- NO precursor (L-arginine): 2,000 mg daily

L-arginine was administered at this dose to enhance nitric oxide bioavailability. Prior studies have shown that doses between 1,500 and 2,500 mg/day improve endothelial function and cerebral blood flow in stroke patients.

Supplementation was provided in the form of oral capsules packaged in identical containers to maintain blinding. Compliance with supplementation was monitored through monthly pill counts and patient adherence logs, ensuring treatment fidelity.

This dosage selection was guided by prior experimental and clinical research on antioxidant therapy in stroke and neurovascular diseases. The aim was to provide effective antioxidant support without exceeding safe and clinically established thresholds.

### Clinical assessments

- Stroke Severity: Stroke severity was assessed at baseline (within 72 hours of stroke onset) using the National Institutes of Health Stroke Scale (NIHSS). The NIHSS is a validated tool for quantifying stroke severity based on neurological examination. Higher scores indicate more severe impairment.
- Functional Outcome: Recovery and functional outcomes were measured using the modified Rankin Scale (mRS) at baseline and at follow-up visits 2, 4, and 6 months post-treatment. The mRS is a widely used scale to assess the degree of disability or dependence in daily activities, where a score of 0 indicates no symptoms and a score of 6 indicates death.
- Antioxidant Biomarkers: Serum levels of SOD, GSH, Catalase, and NO were measured at baseline and the 6-month follow-up visit. Blood samples were collected from each participant via venipuncture in the morning after a 12-hour fast. The serum was separated by centrifugation and stored at 80°C until analysis.
- ◦ SOD Activity was measured using a colourimetric assay (Cayman Chemical Company, USA), where the reduction of superoxide radicals was detected by monitoring absorbance at 450 nm.
- ◦ GSH Levels were determined using a fluorometric assay (Cayman Chemical Company, USA) based on the reaction of GSH with 5,5’-dithio-bis-2-nitrobenzoic acid (DTNB) to form a coloured product, which was quantified by fluorescence at 420 nm.
- ◦ Catalase Activity was quantified by measuring the decrease in hydrogen peroxide absorbance at 240 nm (Aebi, 1984).
- ◦ NO Levels were measured by determining the concentration of nitrite, a stable end product of NO, using the Griess reaction. The absorbance was measured at 540 nm, and nitrite levels were calculated using a standard curve.

### Statistical analysis

Data were analysed using SPSS version 25 (IBM Corp., Armonk, NY, USA). Descriptive statistics were used to summarise baseline characteristics and biochemical markers. Continuous variables were ex pressed as means ± standard deviations (SD), and categorical variables were presented as frequencies and percentages.

Independent t-tests (for continuous variables) and chi-square tests (for categorical variables) were used to compare differences in demographic characteristics and biochemical markers between the two groups. For longitudinal data analysis (i.e., changes in stroke severity and functional outcomes over time), repeated measures ANOVA was performed.

To assess the relationship between antioxidant levels and stroke severity, Pearson correlation coefficients were calculated for each antioxidant marker with NIHSS and mRS scores at baseline, 2 months, 4 months, and 6 months. The relationship between antioxidant levels and recovery outcomes was assessed using linear regression, adjusting for potential confounders (e.g., age, gender, baseline stroke severity, comorbidities).

A p-value of <0.05 was considered statistically significant.

### Ethical considerations

This study adhered to the ethical principles outlined in the Declaration of Helsinki and was conducted in compliance with local regulations and ethical guidelines. Participants’ confidentiality was maintained, and all personal identifiers were removed from the dataset during analysis. Participants were free to withdraw from the study at any time without any impact on their medical care.

## Results

### Participant characteristics

A total of 364 patients participated in the study: 193 patients in Group A (control group) and 171 patients in Group B (experimental group). The mean age of the participants was 67.3±12.2 years, with 203 males (55.7%) and 161 females (44.3%). There were no significant differences in baseline demographic characteristics between the two groups, including age, gender, and comorbidities (p>0.05). Table 1 summarises these characteristics.

**Table 1:** Baseline demographic and clinical characteristics.

Characteristic	Group A<br>(n=193)	Group B<br>(n=171)	p-value
Age (years)	67.5±12.3	67.0±12.1	0.594
Gender			
Male (%)	109 (56.5%)	94 (55.0%)	0.753
Female (%)	84 (43.5%)	77 (45.0%)	
Comorbidities			
Hypertension (%)	122 (63.2%)	106 (61.8%)	0.805
Diabetes (%)	63 (32.6%)	58 (33.9%)	0.773
NIHSS Score	14.7±6.5	14.2±6.0	0.438
mRS Score	4.2±1.1	4.0±1.2	0.324

### Stroke severity at baseline

At baseline, the mean NIHSS score for the cohort was 14.5±6.3, indicating moderate to severe stroke severity. No significant differences were observed between the two groups (Group A: 14.7±6.5; Group B: 14.2±6.0; p=0.438). Similarly, the mean baseline mRS score for the cohort was 4.1±1.2, with no significant difference between groups (Group A: 4.2±1.1; Group B: 4.0±1.2; p=0.324).

### Antioxidant marker levels at baseline and 6-month follow-up

Serum levels of antioxidant markers (SOD, GSH, Catalase, and NO) were measured at baseline and after 6 months of treatment. Table 2

- SOD (Superoxide Dismutase): At baseline, SOD levels did not differ significantly between the groups (Group A: 13.4±4.2 U/mL, Group B: 13.5±4.5 U/mL, p=0.912). However, after 6 months, SOD levels were significantly higher in Group B (16.3±3.7 U/mL) compared to Group A (12.5±4.1 U/mL, p=0.014).
- GSH (Glutathione): Baseline GSH levels were comparable between groups (Group A: 149±28 μmol/L; Group B: 150±30 μmol/L; p=0.856). After 6 months, Group B exhibited significantly higher GSH levels (178±31 μmol/L) than Group A (145±26 μmol/L, p=0.032).
- Catalase: Baseline catalase levels did not differ between groups (Group A: 49.8±10.1 U/mL; Group B: 49.4±9.7 U/mL; p=0.824). At 6 months, no significant difference in catalase levels was observed between the groups (Group A: 49.6±10.2 U/mL; Group B: 52.3±11.1 U/mL; p=0.213).
- NO (Nitric Oxide): Baseline NO levels were similar between groups (Group A: 37.8±7.2 μmol/L; Group B: 38.4±7.9 μmol/L; p=0.681). However, after 6 months, NO levels were significantly higher in Group B (42.1±8.6 μmol/L) compared to Group A (35.4±7.3 μmol/L, p=0.021).

**Table 2:** Antioxidant marker levels at baseline and 6-month follow-up.

Marker	Group A Baseline	Group B Baseline	Group A 6-Month	Group B 6-Month	p-value (6 months)
SOD (U/mL)	13.4±4.2	13.5±4.5	12.5±4.1	16.3±3.7	0.014
GSH (μmol/L)	149±28	150±30	145±26	178±31	0.032
Catalase (U/mL)	49.8±10.1	49.4±9.7	49.6±10.2	52.3±11.1	0.213
NO (μmol/L)	37.8±7.2	38.4±7.9	35.4±7.3	42.1±8.6	0.021

### Stroke severity and functional outcomes

Stroke severity and recovery were assessed by NIHSS and mRS scores at baseline and follow-up at 2, 4, and 6 months. Table 3

- NIHSS Scores: At 2 months, Group B exhibited a significant reduction in NIHSS scores compared to Group A (Group A: 9.6±4.3; Group B: 7.4±3.6, p=0.015). This trend continued at 4 months (Group A: 7.3±3.1; Group B: 5.2±2.9, p=0.010) and 6 months (Group A: 6.1±2.8; Group B: 4.0±2.2, p=0.003), indicating better recovery in the antioxidant supplementation group.
- mRS Scores: At 2 months, Group B had a significant improvement in functional recovery compared to Group A (Group A: 3.7±1.0; Group B: 3.1±0.9; p=0.027). The improvement continued at 4 months (Group A: 3.1±0.8; Group B: 2.5±0.7; p=0.015) and 6 months (Group A: 2.9±0.7; Group B: 2.1±0.6; p=0.003).

**Table 3:** Stroke severity (NIHSS) and functional outcome (mRS) scores over time.

Timepoint	Group A NIHSS	Group B NIHSS	p-value (NIHSS)	Group A mRS	Group B mRS	p-value (mRS)
Baseline	14.7±6.5	14.2±6.0	0.438	4.2±1.1	4.0±1.2	0.324
2 Months	9.6±4.3	7.4±3.6	0.015	3.7±1.0	3.1±0.9	0.027
4 Months	7.3±3.1	5.2±2.9	0.010	3.1±0.8	2.5±0.7	0.015
6 Months	6.1±2.8	4.0±2.2	0.003	2.9±0.7	2.1±0.6	0.003

### Correlations between antioxidant levels and stroke severity

Pearson correlation analyses revealed that higher levels of SOD, GSH, and NO were inversely correlated with NIHSS scores at baseline and 6 months, suggesting an association between these antioxidants and less severe stroke symptoms.

SOD: A significant negative correlation was observed between SOD levels and NIHSS scores at baseline (r=-0.215, p=0.002) and at 6 months (r=-0.295, p=0.001).

GSH: GSH levels were also inversely correlated with NIHSS scores at baseline (r=-0.186, p=0.004) and at 6 months (r=-0.253, p=0.003).

NO: NO levels showed a similar inverse relationship with NIHSS scores at baseline (r=-0.228, p=0.001) and at 6 months (r=-0.272, p=0.002).

Catalase: No significant correlation was found between catalase levels and NIHSS scores at baseline (r=-0.029, p=0.742) or at 6 months (r=-0.046, p=0.617).

### Recovery outcomes and antioxidant levels

At 6 months, higher levels of SOD, GSH, and NO were significantly associated with improved recovery, as measured by mRS scores.

- SOD: Higher levels of SOD were inversely correlated with mRS scores at 6 months (r=-0.271, p=0.002).
- GSH: Similarly, GSH levels were negatively correlated with mRS scores at 6 months (r=-0.198, p=0.006).
- NO: NO levels were also inversely correlated with mRS scores at 6 months (r=-0.234, p=0.004).
- Catalase: No significant correlation was found between catalase levels and mRS scores at 6 months (r=-0.031, p=0.736).

### Adverse events and compliance

No serious adverse events related to antioxidant supplementation were reported, and adherence to the supplementation regimen was high, with over 90% of participants in Group B reporting full compliance.

The results demonstrate that higher levels of SOD, GSH, and NO are significantly associated with reduced stroke severity and improved functional recovery in ischemic stroke patients. In contrast, catalase did not show a significant association with either stroke severity or recovery outcomes. These findings suggest that SOD, GSH, and NO may play a pivotal role in stroke recovery and could serve as potential biomarkers for prognosis and targets for therapeutic intervention.

## Discussion

This study aimed to investigate the association between antioxidant markers (SOD, GSH, NO, and Catalase) and ischemic stroke severity and recovery outcomes in a cohort of 364 patients. The results suggest that higher levels of SOD, GSH, and NO are significantly associated with less severe stroke outcomes and better recovery, while catalase did not show any substantial correlation with stroke severity or recovery. These findings underscore the importance of oxidative stress in the pathophysiology of ischemic stroke and suggest potential therapeutic implications for antioxidant supplementation.

Oxidative stress is a well-established mechanism in the pathogenesis of ischemic stroke, and antioxidants play a crucial role in mitigating oxidative damage [23]. The brain, due to its high metabolic activity and limited endogenous antioxidant defence mechanisms, is particularly vulnerable to the damaging effects of reactive oxygen species (ROS) during ischemia. SOD, GSH, NO, and catalase are integral to the body’s antioxidant defence systems, and their serum levels have been investigated as potential biomarkers for stroke severity and recovery [24].

Our results support previous studies suggesting that antioxidants such as SOD, GSH, and NO may be protective in ischemic stroke. SOD is a primary antioxidant enzyme responsible for converting superoxide radicals into hydrogen peroxide, which is subsequently detoxified by catalase and GSH. In our study, SOD levels were significantly higher in the experimental group (Group B) compared to the control group (Group A) after 6 months of antioxidant supplementation, and this increase was inversely correlated with stroke severity as measured by NIHSS. This finding is consistent with earlier studies that have shown that higher levels of SOD are associated with less neuronal damage and better outcomes in both animal models and human stroke patients [13] [25] [26]. The association between higher SOD levels and improved functional recovery (as measured by mRS scores) further strengthens the potential therapeutic value of this enzyme in the management of ischemic stroke.

Similarly, glutathione (GSH), a key intracellular antioxidant, plays a vital role in neutralising ROS and maintaining cellular redox homeostasis. GSH levels were significantly higher in the experimental group. They were inversely correlated with both NIHSS and mRS scores, indicating that higher GSH levels were associated with less severe strokes and better recovery. This aligns with findings from other clinical studies that have shown a positive relationship between elevated GSH levels and improved stroke recovery outcomes [15] [27] [28]. GSH’s role in reducing lipid peroxidation and preserving mitochondrial function makes it an attractive candidate for therapeutic strategies aimed at limiting oxidative damage after ischemic stroke.

Nitric oxide (NO) is another antioxidant that has received increasing attention for its neuroprotective properties in ischemic stroke. In addition to its well-established role as a vasodilator, NO also helps reduce apoptosis and modulates blood-brain barrier integrity during ischemic injury [29]. In our study, NO levels were significantly higher in the experimental group and were inversely correlated with stroke severity (NIHSS) and functional impairment (mRS), suggesting a potential protective role in stroke recovery. While the precise mechanisms through which NO exerts neuroprotection remain complex, its ability to reduce ROS levels and regulate inflammatory responses likely contributes to improved recovery outcomes in stroke patients.

However, catalase did not show a significant association with stroke severity or recovery, a finding that warrants further exploration. One possible explanation is that while catalase is essential for detoxifying hydrogen peroxide, its role may be more limited in the broader context of ischemic stroke pathology. Unlike SOD and GSH, which directly modulate oxidative stress at multiple levels, catalase primarily acts on hydrogen peroxide, a relatively stable ROS intermediate, rather than on highly reactive oxygen species such as superoxide or peroxynitrite [30]. Additionally, catalase activity is often compensated by other antioxidant systems, particularly glutathione peroxidase, which may explain why serum catalase levels did not correlate strongly with clinical outcomes in our study. This is consistent with other studies that have failed to demonstrate a robust association between catalase activity and stroke severity or recovery [16] [31]. Previous research has also suggested that catalase activity may be more relevant in the early acute phase of stroke rather than during the later recovery phase examined in this study [32]. Further studies are needed to determine whether catalase plays a more time-dependent or localised role in ischemic stroke and whether other enzymatic interactions influence its function.

Žitňanová and colleagues [33] in conducted a study focusing on oxidative stress markers and their dynamic changes in the early stages and up to three months post-acute ischemic stroke (AIS). They found that oxidative damage was highest within 24 hours of stroke onset, with elevated levels of lipid peroxides and antioxidant enzyme activities, such as SOD and catalase. In contrast, our study specifically investigated the relationship between antioxidant markers – SOD, GSH, catalase, and NO – with ischemic stroke severity and recovery outcomes in a larger cohort over 6 months. While Žitňanová et al. [33] focused on temporal changes in oxidative stress markers, our findings highlight those higher levels of SOD, GSH, and NO correlate with less severe stroke symptoms and better recovery, suggesting their role in improving stroke outcomes. Notably, unlike the Žitňanová study, we observed no significant correlation between catalase levels and either stroke severity or recovery, further reinforcing the potential significance of SOD, GSH, and NO in ischemic stroke recovery.

Pinoșanu and colleagues [34] investigated the impact of concurrent COVID-19 infection on oxidative stress and antioxidant defence mechanisms in patients with acute ischemic stroke. They found significantly elevated oxidative stress, mainly through increased levels of TBARS, and enhanced antioxidant activities such as GSH, glutathione peroxidase (GPx), superoxide dismutase SOD, and catalase (CAT) in the Stroke-COVID group compared to those with ischemic stroke alone. This study highlights the amplified oxidative damage and inflammatory response in the context of concurrent stroke and COVID-19, which may contribute to worse outcomes. In comparison, our study focused on the relationship between antioxidant markers (SOD, GSH, NO, and CAT) and ischemic stroke severity and recovery, and we found that higher levels of SOD, GSH, and NO were associated with better recovery and less severe stroke outcomes. While both studies emphasise the role of oxidative stress in ischemic stroke, our study does not examine the added complexity of COVID-19 infection, which may further influence the balance between oxidative damage and antioxidant defence.

The findings of this study have significant implications for the rehabilitation and management of ischemic stroke patients. Higher levels of SOD, GSH, and NO were associated with less severe stroke outcomes and better recovery, suggesting that boosting antioxidant defences could be an effective therapeutic strategy. The significant improvement in recovery seen in the experimental group receiving antioxidant supplementation highlights the potential role of antioxidant therapy in supporting stroke rehabilitation. It is important to note that the experimental group did not receive antioxidant supplementation in isolation but in combination with standard stroke rehabilitation therapy. This further emphasises the potential synergistic effects of antioxidant supplementation in conjunction with conventional treatment.

These findings suggest that antioxidant supplementation may offer an adjunctive approach to current stroke rehabilitation strategies, particularly in patients who have suffered moderate to severe strokes. While the precise mechanism through which antioxidants facilitate recovery remains an area of active research, these compounds likely help reduce oxidative damage, preserve neuronal integrity, and promote neuroplasticity, all of which contribute to functional recovery.

Despite the promising findings, several limitations must be acknowledged. This study was conducted as a single-centre trial, which may limit the generalizability of the results to broader populations with varying genetic, environmental, and healthcare factors. A multicenter study would provide a more representative dataset and enhance external validity. Additionally, while we followed patients for six months, the long-term effects of antioxidant supplementation on stroke recovery remain unclear. Antioxidant-related improvements may extend beyond this period, necessitating future research with extended follow-ups and additional time points to capture biomarker fluctuations throughout recovery better.

Another key challenge is the clinical implementation of SOD, GSH, and NO as biomarkers. Although this study suggests that higher levels correlate with improved outcomes, interpreting borderline or intermediate values remains difficult. Clinicians require clear reference ranges and standardised guidelines to determine meaningful changes in antioxidant status. Furthermore, factors such as diet, medication use, and comorbidities may influence antioxidant levels, complicating their use as routine clinical markers. Future research should focus on defining optimal cutoff values and assessing their predictive accuracy across diverse populations.

This study was not designed to identify the optimal dosage or formulation of antioxidant supplementation, which may vary based on bioavailability and individual responses. Additionally, the lack of a third group receiving antioxidants without rehabilitation limits our ability to isolate the specific contribution of supplementation from standard therapy. Lastly, we did not investigate the molecular mechanisms through which SOD, GSH, and NO exert their effects in ischemic stroke. Future studies should explore their roles in mitochondrial function, neuroinflammation, and apoptosis, providing deeper insight into their therapeutic potential.

## Conclusion

This study demonstrates that higher levels of SOD, GSH, and NO are significantly associated with reduced stroke severity and better recovery outcomes, while catalase did not show a meaningful correlation with clinical outcomes. These findings highlight the importance of oxidative stress in ischemic stroke and suggest that SOD, GSH, and NO could serve as valuable biomarkers for stroke prognosis and therapeutic targets. However, further prospective studies with more extensive multicenter cohorts are necessary to validate these results and establish standardised reference ranges for these biomarkers in clinical practice. Additionally, long-term follow-up beyond six months is required to assess the sustainability of antioxidant effects on stroke recovery, as well as to determine the optimal dosage regimens and independent effects of antioxidant therapy compared to standard rehabilitation.

Clinically, these biomarkers could provide a means for early identification of high-risk patients, enabling personalised interventions that improve neuroprotection and recovery. Targeted antioxidant therapies, once validated, could be incorporated into rehabilitation protocols to enhance functional outcomes in ischemic stroke patients. Further research is needed to explore the molecular mechanisms underlying the antioxidant effects, including their roles in mitochondrial function, neuroinflammation, and neuronal repair. By deepening our understanding of these processes, we may open the door to novel antioxidant-based therapeutic strategies, expanding treatment options and improving clinical management for ischemic stroke patients.

## Dodatak

### Acknowledgements

We would like to express our sincere gratitude to all the participants of this study, without whom this research would not have been possible.

### Funding

This study received no funding.

### Ethical considerations

This study was conducted in accordance with the principles of the Declaration of Helsinki. Informed consent was obtained from all participants prior to enrollment in the study. Participants’ confidentiality was maintained, and all data were anonymised to ensure privacy.

### Data availability

The datasets generated and/or analysed during the current study are available from the corresponding author upon reasonable request.

### Conflict of interest statement

All the authors declare that they have no conflict of interest in this work.
